# Prostate-specific membrane antigen and fibroblast activation protein distribution in prostate cancer: preliminary data on immunohistochemistry and PET imaging

**DOI:** 10.1007/s12149-021-01702-8

**Published:** 2021-12-02

**Authors:** Katharina Kessel, Robert Seifert, Matthias Weckesser, Martin Boegemann, Sebastian Huss, Clemens Kratochwil, Uwe Haberkorn, Frederik Giesel, Kambiz Rahbar

**Affiliations:** 1 Department of Nuclear Medicine, University Hospital Muenster, Albert-Schweitzer-Campus 1, 48149 Münster, Germany; 2 Department of Nuclear Medicine, University Hospital Essen, Essen, Germany; 3 Department of Urology, University Hospital Muenster, Münster, Germany; 4 Gerhard-Domagk-Institute for Pathology, University Hospital Muenster, Münster, Germany; 5 Department of Nuclear Medicine, University of Heidelberg, Heidelberg, Germany; 6 Clinical Cooperation Unit Nuclear Medicine, German Cancer Research Center (DKFZ), Heidelberg, Germany; 7 Translational Lung Research Center Heidelberg (TLRC), German Center for Lung Research (DZL), Heidelberg, Germany; 8 Department of Nuclear Medicine, University Hospital Düsseldorf, 40210 Düsseldorf, Germany

**Keywords:** PSMA, FAP, Fibroblast activation protein, Prostate cancer

## Abstract

**Introduction:**

Fibroblast activation protein (FAP) has been recently presented as new imaging target for malignant diseases and offers high contrast to surrounding normal tissue. FAP tracer uptake has been reported in various tumor entities. The aim of this study was to compare FAP and Prostate-specific membrane antigen (PSMA) expression in primary prostate cancer employing histological analyses and PET imaging in two small patient collectives.

**Methods:**

Two independent small patient collectives were included in this study. For cohort A, data of 5 prostate cancer patients and 3 patients with benign prostate hyperplasia were included. Patients with prostate cancer were initially referred for PSMA PET staging. Radical prostatectomy was performed in all patients and prostate specimen of patients and biopsies of healthy controls were available for further evaluation. Histological workup included HE and immunohistochemistry using PSMA Ab, FAP Ab. Cohort B consists of 6 Patients with diagnosed mCRPC and available PSMA as well as FAP PET.

**Results:**

Patients with proven prostate cancer infiltration exhibited strong positivity for PSMA in both primary tumors and lymph node metastases while stainings for FAP were found positive in some cases, but not all (2/5). Controls with BPH presented moderate PSMA staining and in one case also with a positive FAP staining (1/3). PET imaging with FAP seemed to result in more precise results in case of low PSMA expression than PSMA-PET.

**Conclusions:**

While PSMA staining intensity is a valid indicator of prostate cancer in both primary tumor and lymph node metastases, the expression of FAP seems to be heterogeneous but not necessarily linked to cancer-associated fibroblasts. It is also present in inflammation-associated myofibroblasts. Therefore, its ultimate role in prostate cancer diagnosis remains a subject of discussion.

## Introduction

Cancer-associated fibroblasts (CAFs) are part of the reactive stroma of epithelial tumors. CAFs mediate tumorigenesis and metastasis by secreting tumor cell stimulating factors such as TGFβ as well as through the action of fibroblast activation protein (FAP), thereby facilitating tumor cell migration [1]. FAP overexpression leads to a higher risk of tumor invasion, lymph node metastasis and to decreased overall survival (OS) [2] and has been shown to be expressed in various cancers including prostate cancer, as well as chronic inflammatory diseases with fibrotic changes [3]. Absent in normal healthy adult tissue, FAP is normally expressed during development and highly upregulated at sites of active tissue remodeling, such as wound healing, fibrosis and cancer [4, 5]. Over 90% of epithelial tumors express FAP, including prostate cancer [3, 6, 7]. In most tumor types, FAP expression is associated with increased lymph node metastasis and decreased overall survival in various tumors [2, 8]. Overexpression of FAP leads to upregulation of proliferation and secretion of inflammatory and extracellular matrix remodeling factors and may even promote angiogenesis [9]. Increased levels of matrix-metalloproteinases in the extracellular matrix (ECM) surrounding CAFs point at migration and invasion and thus promotion of tumorigenic cell behavior [8]. However, this role might vary between different tumors as shown for gastric and lung cancer [10, 11]. In terms of prostate cancer, the role of FAP has not been fully investigated yethowever it seems to have potentially beneficial features for diagnosis through imaging in prostate-specific membrane antigen (PSMA)-negative or -low disease.

Quinoline based fibroblast activation protein inhibitors (FAPIs) have been recently presented as a new imaging tool for malignant diseases, with high contrast to surrounding tissue [3, 12–14]. A range of tracer uptake has been reported for various tumors and recommended as a complementary diagnostic element in addition to ^18^F-FDG-PET [15, 16].

However, little is known about the cancer stage-specific expression patterns of FAP in prostate disease due to a small number of patients that already underwent FAPI-PET imaging. In this study we aimed to investigate the usefulness of FAP as a target for immunohistochemistry (IHC), imaging and subsequent theranostic approaches in prostate cancer besides PSMA. Using a small, heterogeneous collection of PCa cases from two German centers we explore the relevance of FAP as a possible player during disease progression and its role for diagnosis. A total of ten patients was retrospectively enrolled into this study, which creates a preliminary picture and may present the onset of more detailed investigations.

## Methods

### Patient cohorts A and B

Cohort A from Münster comprises of 5 patients with prostate cancer at initial diagnosis and 3 patients with benign prostate hyperplasia (BPH) as controls. The average age was 67 years (49.3–77.6) (see Table 1). Patient tissue biopsies of cohort A of affected prostate and lymph nodes were analyzed by IHC for the expression of PSMA and FAP. Biopsies of control patients were treated and analyzed correspondingly. All five PCA patients of cohort A were referred to PSMA-PET.

**Table 1:** Patient characteristics cohort A

Patient	Age	PE	Gleason score	PSA at PSMA-PET	SUV_max_
A1	73	y	4 + 5 = 9	29.3	5.8
A2	78	y	4 + 3 = 7b	2.41	4.8
A3	60	y	4 + 3 = 7b	12.1	50.3
A4	75	y	5 + 4 = 9	4.8	5.6
A5	49	y	4 + 3 = 7b	28.2	50.5

Only selected parameters are listed

*PE* prostatectomy, *TNM* tumor, lymph nodes, metastasis, *PSA* prostate-specific antigen, *PSMA* prostate-specific membrane antigen, *PET* positron emission topography, *SUV*_*max*_ maximum standard uptake value

Cohort B consists of 6 patients from Heidelberg with a mean age of 70 years (57–78) (Table 2) and with mCRPC. Two of them had neuroendocrine dedifferentiation, which was earlier verified by tissue biopsies (Patients ID: B2, B5). Two patients presented with BRCA mutations (Patient IDs B1 with BRCA ½ WT, B2 with BRCA2). All six patients of cohort B have been subjected to PSMA-PET as well as ^68^Ga-FAPI-PET but not to FAPI-IHC.

**Table 2:** Patient characteristics cohort B

Patient	Age	PE	Gleason score	PSA at PSMA-PET	PSMA SUV_max_	FAPI SUV_max_
B1	67	n	8	85.0	14.4	8.7
B2	64	y	9	11.4	19.9	27.8
B3	76	n	9	3.0	3.2	5.7
B4	57	n	7	169.21	4.3	8.2
B5	76	n	8	0.44	41.8	18.2
B6	78	y	9	36.19	18.3	5.85

Only selected parameters are listed

*PE* prostatectomy, *PSA* prostate-specific antigen, *PSMA* prostate-specific membrane antigen, *PET* positrone emission topography, *SUV*_*max*_ maximum standard uptake value *FAPI* fibroblast activation protein inhibitor

### PSMA and FAP imaging procedure

^18^F-PSMA-1007 was produced in a GE TracerLab MX synthesizer according to the one-step procedure described by Cardinale et al*.* and standard operation procedure described before [17–20].

Patients received 4 MBq per kg body weight with a maximum of 400 MBq per patient. Scanning was performed 120 min p.i. covering lower limbs to the vertex. Imaging at 120 min was previously described to be the optimal timepoint for ^18^F-PSMA-1007 due to a higher contrast (tumor to background ratio) (3).

Patients in cohort B received a ^68^Ga-FAPI-46 and ^68^Ga-PSMA-11 PET/CT [19]. ^68^Ga-FAPI-46 and ^68^Ga-PSMA-11 were produced according to institutional standard procedures guidelines as described before [14]. Patients received a mean of 207,93 MBq of ^68^Ga-FAPI-46 and 221,31 MBq of ^68^ Ga-PSMA-11. Image acquisition for ^68^Ga-FAPI-46 started 10 min p.i. and for ^68^Ga-PSMA-11 started at 60 min p.i. covering lower limb to the vertex.

Patients were asked to void the bladder before the scan. Images were acquired with a scan time of 3 min per bed position on a Siemens mCT scanner (Siemens Healthcare, Knoxville, Tennessee, USA). Image reconstruction was performed using standard manufacturer software. For attenuation correction, a low dose CT was performed accordant to PET images [19, 20].

Image analysis was performed using manufacturers standard software (Syngo-Via, Siemens Healthineers, Knockville, TN). Maximum standard uptake value (SUVmax) was measured in all patients and is presented for the lesion with the highest uptake.

### Immunohistochemistry

Immunohistochemistry (IHC) was performed on 4-µm-thick paraffin sections of prostate tissue using the peroxidase-conjugated avidin–biotin method. Staining was performed fully automated on a Bench Mark Ultra and detected using the OptiViev DAB IHC detection kit (Ventana by Roche, Germany). Antibodies included a monoclonal mouse anti-FAP antibody (ab207178, Roche Ventana, 1:100 dilution). PSMA as well as FAP expression was determined semi-quantitatively, which is a general proceeding of our pathologists. For a positive result at least 10% of the cells had to stain positive.

## Results

**Fig. 1:**
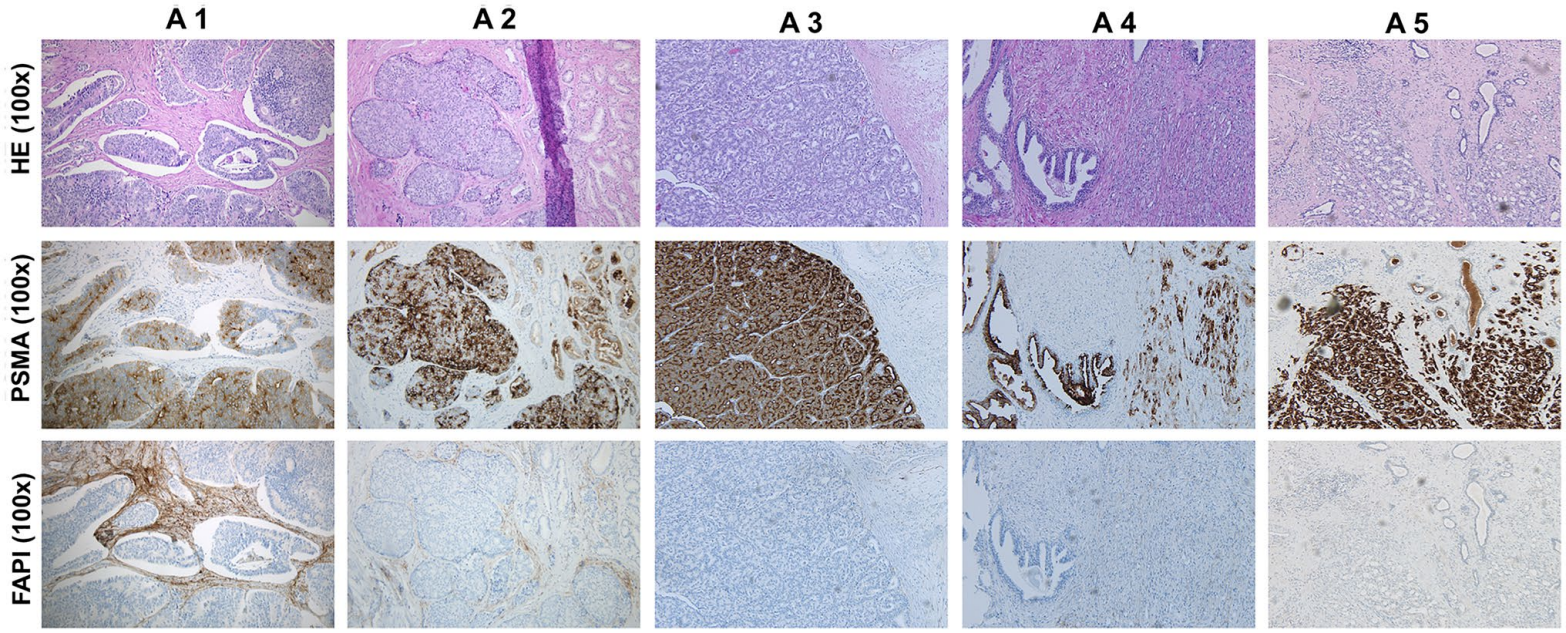
conventional hematoxilineosin and immunohistochemical stainings of PSMA and FAP expression in patients with newly diagnosed prostate cancer. Tissue biopsies were taken at prostatectomy and subsequently subjected to pathological stainings with HE, anti PSMA and anti-FAP. *PSMA* prostate-specific membrane antigen, *FAPI* fibroblast activation protein inhibitor, *HE* hematoxicillin eosin

All five PCA patients of cohort A present with positive PSMA-PET findings. Two patients (ID A1 and A5) presented with lymph node metastases that showed PSMA-positive staining Gleason score 9 and 7, respectively), three patients (ID A2, A3 and A4, Gleason 7,7 and 9, respectively) did not have any reported metastases, thus lymph nodes were PSMA negative. PSMA stainings of prostate specimen were positive in all 5 patients. Only patient A1 displayed positive FAP expression in prostate tissue and in LN metastasis, determined by semi-quantitative measure. Patient A2 showed FAP expression in prostate cancer tissue, but not in LN specimen. Patient A3 and A4 had no expression of FAP in prostate tumor nor LN. Patient A5 was found positive for PSMA in both prostate and LN (not shown), but negative for FAP in both tissues (Fig. 1). Anti-FAP staining exclusively revealed fibroblast-like morphology. An anti-CD163 staining for the detection of M2 macrophages has not been performed. Cohort A had an average PSMA SUVmax of 23.4 measured in prostate tissue at PSMA PET imaging (range 4.8–50.5) (Table 1).

**Fig. 2:**
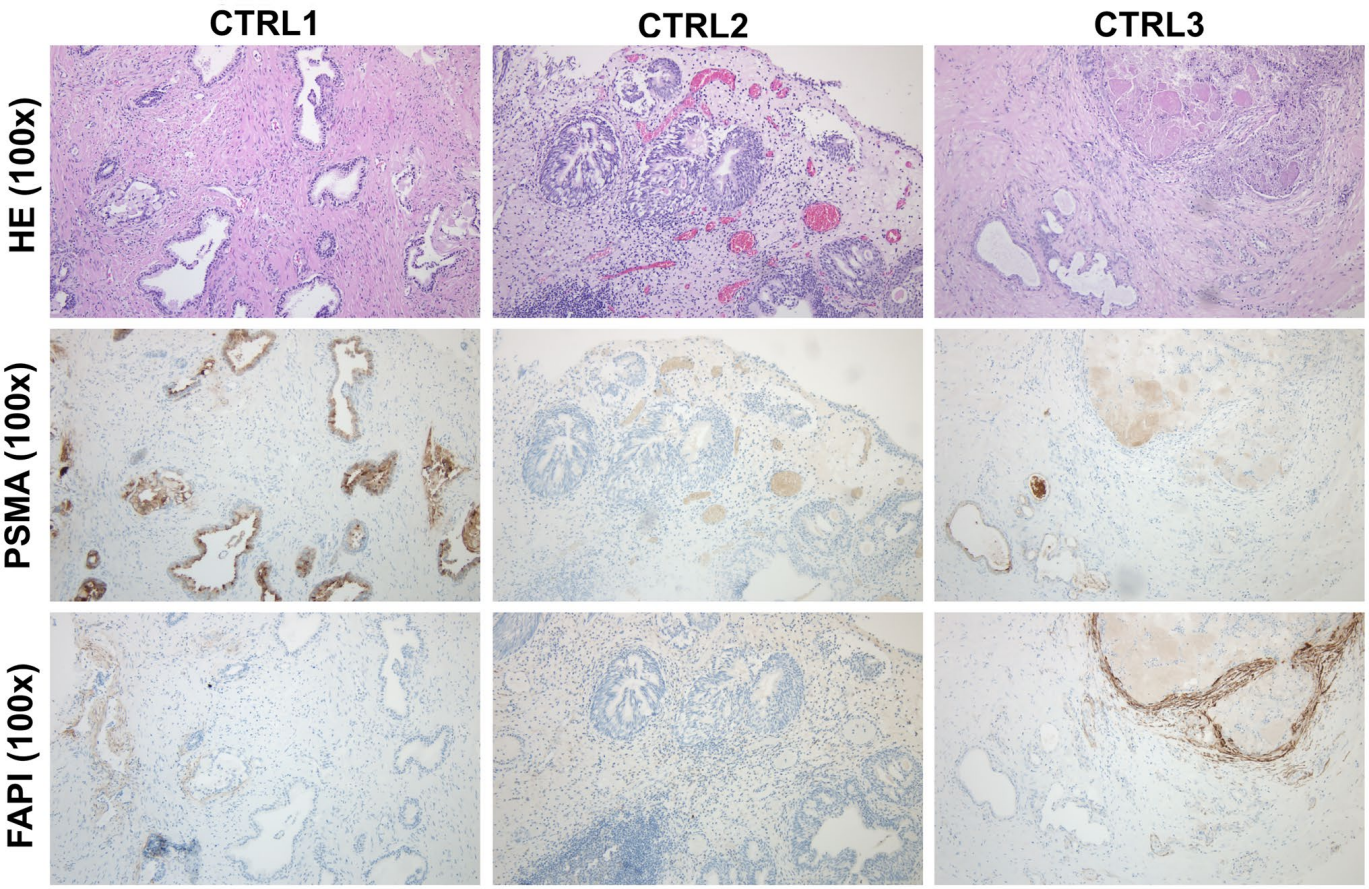
Hematoxilineosin and immunohistochemical stainings of PSMA and FAP expression in control patients with benign prostate hyperplasia. Tissue biopsies were taken and subsequently subjected to pathological stainings with HE, anti PSMA and anti-FAP. *PSMA* prostate-specific membrane antigen, *FAPI* fibroblast activation protein inhibitor, *HE* hematoxicillin eosin, *CTRL* control patient

Control patients presented with overall moderate PSMA expression and accumulation in hyperplastic prostate tissue. 2 out of 3 controls were negative for FAP, while control 3 displayed FAP expression in the stroma surrounding hyperplastic tissue most likely linked to FAP-positive myofibroblasts (Fig. 2).

**Fig. 3:**
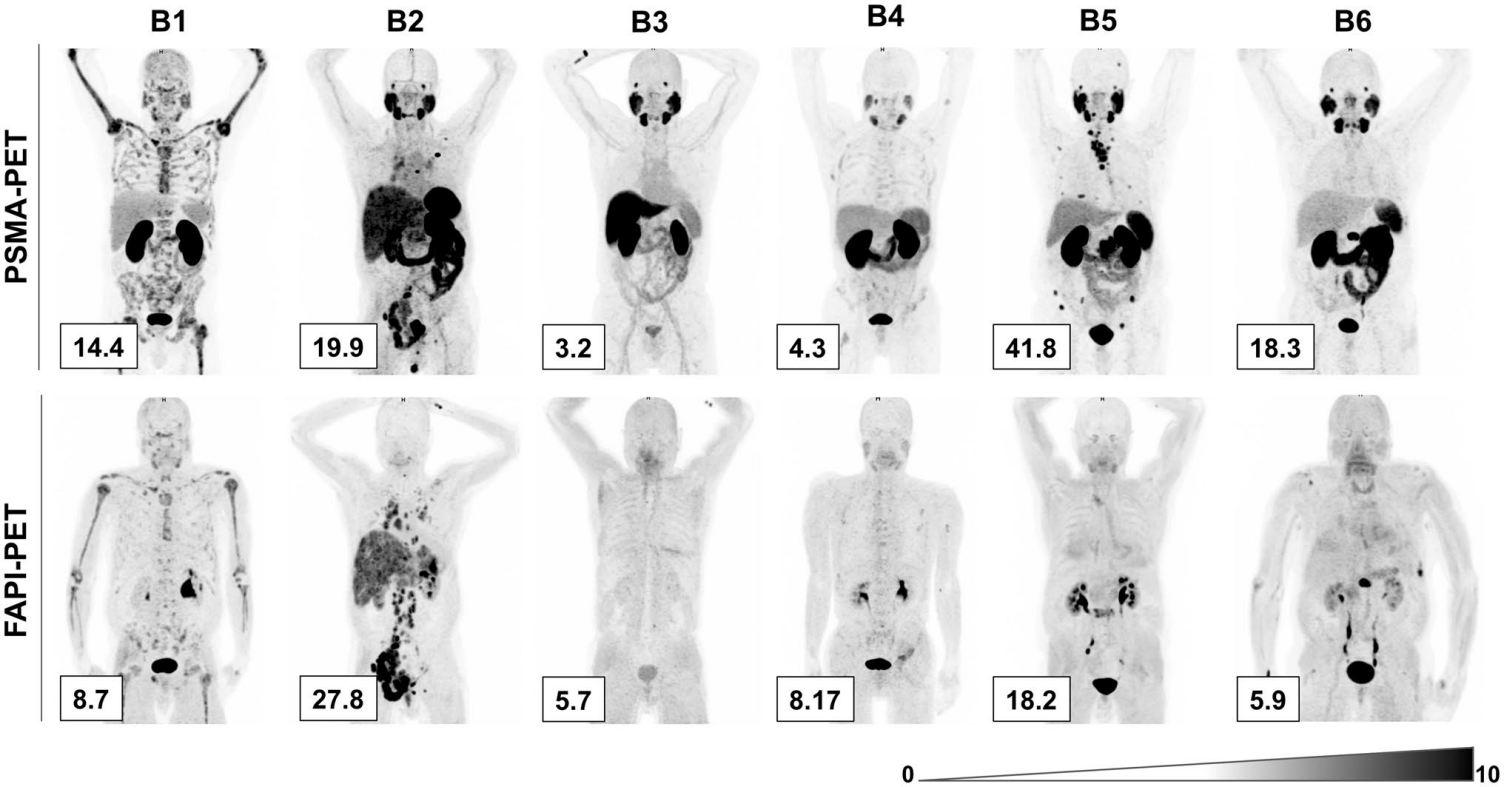
PSMA- and FAP-imaging of cohort B. Patients of cohort B underwent PSMA-PET imaging (upper row) as well as FAPI-PET imaging (lower row). SUV_max_ values are indicated in the lower left corner of each scan. *PSMA* prostate-specific membrane antigen, *FAPI* fibroblast activation protein inhibitor, *SUV*_*max*_ standard uptake value maximum, *PET* positron emission topography

**Fig. 4:**
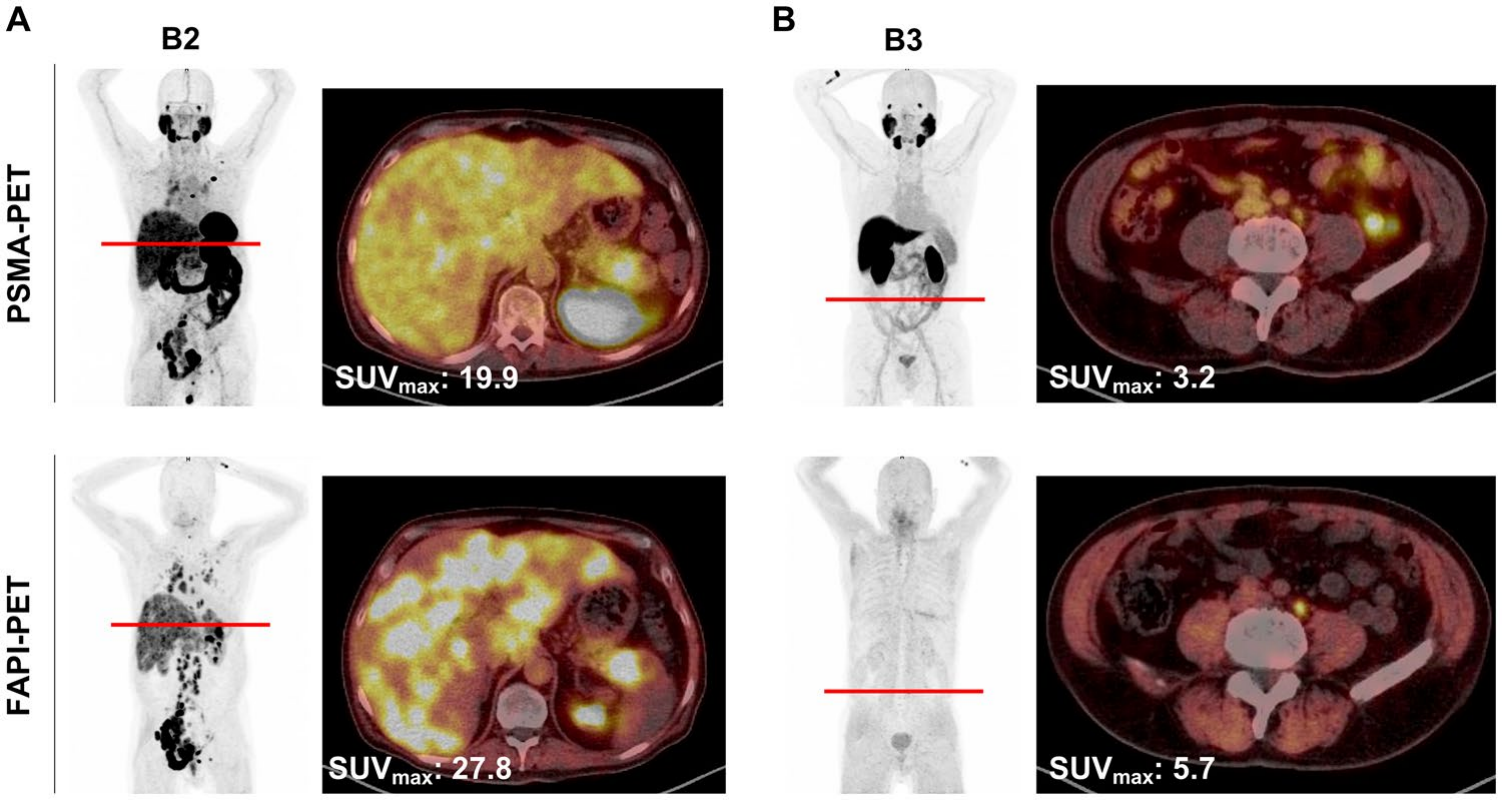
Whole body scans and axial images of cohort B patients. Patients with the highest (B2) and lowest (B3) SUVmax in PSMA scans were selected. Whole body scan and axial images are shown. Red lines indicated selected axis level. White numbers indicate SUV_max_ of the respective scan. *PSMA* prostate-specific membrane antigen, *FAPI* fibroblast activation protein inhibitor, *SUV*_*max*_ standard uptake value maximum, *PET* positron emission topography

Patient B1 carried the BRCA ½ WT mutation, patient B6 carried BRCA2 (Gleason score 8). Patient B2 of cohort B additionally had a neuroendocrine variant of prostate cancer with diffuse metastases of the liver (Gleason score 9). Patient B5 was diagnosed with neuroendocrine differentiation (Gleason score 8). All patients of cohort B underwent PSMA- as well as FAPI-PET/CT (Fig. 3). For selected patients (B2 and B3), axial scans were prepared to compare PSMA and FAP imaging directly and identify questionable lesions from whole body scan images (Fig. 4 Gleason score 9, both). Patient B2 had more FAP positive lesions than PSMA positive ones and had a higher SUVmax, while patient B3 had fewer FAP-positive lesions compared to PSMA PET, yet FAP PET obtained a higher SUVmax compared to PSMA-PET.

## Discussion

Because of its very recent advent in cancer diagnostics, FAP is not yet routinely used to identify cancerous tissue alterations in PC biopsies [12, 21]. Although FAP has been reported to be a useful target for diagnostics and therapy in various cancers, its use in prostate cancer seems however debatable [3]. In the present study, the use of FAP-based histopathological examination of malignant and benign prostate specimen as well as FAP-PET imaging in patients with prostate cancer have been investigated in two small patient collectives.

The nature of FAP expression does not only relate to tumor cell growth and migration, but also to chronic inflammation, such as rheumatoid arthritis, heart infarction and fibroses as well as wound healing, making it rather unspecific in a mixed disease setting and at early diagnosis [22–25]. Recent studies and case reports, however, postulate FAP-positive PET/CT in PSMA negative/FDG positive disease, underlining the relevance of FAP-based imaging only in late stages and clinical aggressive tumors of the prostate [7, 26, 27]. In these studies, FAPI-PET performed better than PSMA-PET in advanced disease [27]. Corroborating this idea, the present study indicates the use of FAP-based diagnostics only in advanced metastatic prostate cancer or in case of doubtful lesions, therapy failure and suspected other primary tumors [3].

However, all patients in this study presented with a Gleason score > 7 and although it seems reasonable to consider FAP expression from this stage and higher, this study did not reveal any link between FAP expression and Gleason score which is probably due to the limited number of cases.

Remarkably, FAP was found in the stroma of BPH in one of the control patients, comparable to FAP expression in cancerous prostate tissue, determined semi-quantitatively. This may hint at chronic prostatitis and represents an inflammatory condition. Although it seems interesting to track the long-term changes in this particular control patient, if it might progress into a cancerous lesion, this situation reflects the current uncertain prognostic value of FAP expression in early prostate disease.

It is known that FAP mediates tumor cell proliferation in gastric cancer via different pathways and thus enables metastasis formation by remodeling the tumor microenvironment [28]. Furthermore, CAFs have been shown to improve the tumor cell invasion and migration by inducing endothelialmesenchymal transition (EMT) and thereby induce a more aggressive phenotype in a mouse model [29]. Compared to normal fibroblasts CAFs overexpress TGFβ and other proteins and secrete IL-22 into the tumor microenvironment which promotes cell invasion via STAT3 and ERK pathways in gastric cancer [30, 31]. Baring this potential, FAP might also play a role in more advanced de-differentiated tumors of the prostate, such as those with neuroendocrine signatures and loss of PSMA expression [32]. In case of prostate cancer FAP might, therefore, aid to proper down- or upstaging of tumors. By this, PSMA-negative disease and single dubious lesions might be diagnosed at higher precision. However, this retrospective study supports this insufficiently due to the heterogeneity and small number of the patient collective and type of data collection. Nonetheless, in the light of the deficient data availability on FAP function in prostate cancer this study represents a first glimpse at the putatively complex interactions.

General findings regarding the tumor promoting function of CAFs most likely apply for prostate cancer as well as for any other cancer typehowever specific interactions have not yet been reported. , it is currently unknown at which stage exactly FAP expression evolves in prostate cancer and to which extend it correlates to tumor load and aggressiveness, although a positive correlation of FAP expression has been detected with advancing prostate cancer, being highest im CRPC [32]. Therefore, diagnostic use of FAP-based imaging might be recommended more as a complementary tool in later stages of prostate cancer together with PSMA and/or FDG PET as well as DOTATATE-PET for specifically targeting developing or established neuroendocrine de-differentiation.

We recently have taken first steps by analyzing evolving neuroendocrine gene expression signatures in CTCs of PCA patients, yet there is still need to investigate correlations between gene expression and imaging, especially targeted on progressing tumors [33]. Novel imaging techniques such as FAP-based imaging should be included in these analyses. FAP might outperform the commonly used FDG-PET due to less requirements for its application and less unspecific noise due to abundant glucose metabolism. In rectal cancer FAP expression in CAFs correlates with poor prognosis after chemoradiotherapy [34]. Therefore, FAPI uptake may indicate active tumors with tendencies of progression and differentiation towards a more aggressive stage. Its use might enable a new kind of tumor development monitoring to predict if a tumor will spread and where potential metastases will appear due to a FAP positive environment. However, if that holds promise and how FAP expression will be scaled and which cutoffs define inflammation and malign conditions is still a matter of future investigations.

Investigations using FAP-based imaging and DOTATATE-PET would definitely clarify its association with neuroendocrine differentiation. Double IHC stainings with anti-FAP and anti-SSR would be another diagnostic tool to support this strategy. Studies to investigate this interconnection are urgently needed, especially with regard to specific therapies of PSMA low or negative patients which had poor outcomes and were ineligible for PSMA targeted therapy [35]. Although PSMA-RLT is a promising treatment in advanced, PSMA positive mCRPC [36], tumors that loose PSMA expression need a more tailor-made therapy based on their specific signatures. FAP imaging represents a reasonable tool to identify those heterogeneous tumors and might aid to choose the optimal therapy setup and target. A tandem therapy using PSMA and FAP targeted radionuclides or a combination of radionuclide therapy and other biotherapeutics might be of interest in future studies [37].

In case of PSMA negative disease, the use of FAP could be a promising replacement for FDG-PET, although it is still unknown, whether all PSMA negative tumors develop corresponding homogeneous expression of FAP. Immunohistochemical detection of FAP expression in tumor tissue and imaging might nonetheless point at progression of an early stage to a more aggressive disease. Furthermore, FAP targeted imaging aids to reveal the presence of additional primary tumors as has been shown in a case of newly diagnosed PSMA-negative metastatic gastric signet-ring cell carcinoma in a patient with known PSMA-positive prostate cancer [15]. Further, persistent FAP expression in inflammatory environments such as BPH and prostatitis might offer options to observe and detect malign development in realtime and non-invasively.

Our study is a first step towards the combination of analyzing FAP expression with different technologies. The two patient cohorts analyzed here cannot be combined as one due to the different type of retrospective data generated herein. However, two hypotheses that have to be investigated further evolved: first, FAP expression alone may be not useful for early and initial diagnosis of prostate cancer as it is unspecific and found in multiple conditions. Second, FAP can be useful for later disease stages, especially in case of PSMA negative disease or heterogeneous PSMA expression. Further, FAP aids to detect other primary tumors of different origin. We recently successfully investigated the usefulness of alternating targeted therapies such as local SIRT of the liver in combination with PSMA-RLT [38]. In terms of FAP as a target, such a therapy strategy is very likely to be of similar benefit for patients undergoing PSMA-RLT, who present with heterogeneous PSMA expression.

As up to date only few patients underwent FAP-PET, it might be too early to draw conclusions about the usefulness of FAP-PET in prostate cancer. However, recently published case series and studies reveal promising results to use it as an additional diagnostic tool. This study aimed at generating preliminary data as a foundation for future prospective approaches for the safe application of FAP-based diagnostics in prostate cancer.

## Conclusions

The expression of FAP in prostate disease settings, tumorous as well as inflammatory seems to not necessarily linked to cancer-associated fibroblasts but also expressed by benign myofibroblasts in suspected chronic prostate inflammation. This hampers the diagnostic relevance of FAP in early prostate cancer. By considering and including FAP expression into a diagnostic panel, physicians might be able to discriminate between responding and progressive patients. However, the role of FAP in prostate cancer diagnosis remains subject to further investigations.
